# Efficacy and safety of second-line cabozantinib after immuno-oncology combination therapy for advanced renal cell carcinoma: Japanese multicenter retrospective study

**DOI:** 10.1038/s41598-023-48087-4

**Published:** 2023-11-23

**Authors:** Tomokazu Sazuka, Yuto Matsushita, Hiroaki Sato, Takahiro Osawa, Nobuyuki Hinata, Shingo Hatakeyama, Kazuyuki Numakura, Kosuke Ueda, Takahiro Kimura, Masayuki Takahashi, Hajime Tanaka, Yoshihide Kawasaki, Toshifumi Kurahashi, Takuma Kato, Kazutoshi Fujita, Makito Miyake, Takahiro Kojima, Hiroshi Kitamura, Hideaki Miyake, Tomohiko Ichikawa

**Affiliations:** 1 Department of Urology, Chiba University Graduate School of Medicine, 1-8-1 Inohana, Chuo-ku, Chiba, 260-8670 Japan; 2 Department of Urology, Hamamatsu University School of Medicine, Hamamatsu, Japan; 3 Department of Urology, Hokkaido University Hospital, Sapporo, Japan; 4 Department of Urology, Hiroshima University Graduate School of Biomedical Sciences, Hiroshima, Japan; 5 Department of Advanced Blood Purification Therapy, Hirosaki University Graduate School of Medicine, Hirosaki, Japan; 6 Department of Urology, Akita University Graduate School of Medicine, Akita, Japan; 7 Department of Urology, Kurume University School of Medicine, Kurume, Japan; 8 Department of Urology, The Jikei University School of Medicine, Tokyo, Japan; 9 Department of Urology, Tokushima University Graduate School of Biomedical Sciences, Tokushima, Japan; 10 Department of Urology, Tokyo Medical and Dental University, Tokyo, Japan; 11 Department of Urology, Tohoku University Graduate School of Medicine, Sendai, Japan; 12 Department of Urology, Hyogo Prefectural Cancer Center, Akashi, Japan; 13 Department of Urology, Faculty of Medicine, Kagawa University, Takamatsu, Japan; 14 Department of Urology, Faculty of Medicine, Kindai University, Osaka, Japan; 15 Department of Urology, Nara Medical University, Kashihara, Japan; 16 Department of Urology, Aichi Cancer Center, Nagoya, Japan; 17 Department of Urology, University of Toyama, Toyama, Japan

**Keywords:** Cancer, Medical research, Molecular medicine, Oncology, Risk factors, Urology

## Abstract

Immuno-oncology (IO) combination therapy is utilized as a first-line systemic treatment for advanced renal cell carcinoma. However, evidence supporting the use of cabozantinib after IO combination therapy is lacking. We retrospectively analyzed patients who received second-line cabozantinib after IO combination therapy using the Japanese Urological Oncology Group (JUOG) database. In total, 254 patients were enrolled in the JUOG global study, and 118 patients who received second-line cabozantinib comprised the study cohort. The objective response rate, disease control rate, second-line cabozantinib progression-free survival (PFS), and overall survival from second-line for overall were 32%, 75%, 10.5 months, and not reached, respectively, for first-line IO-IO therapy were 37%, 77%, 11.1 months, and not reached, respectively, versus 24%, 71%, 8.3 months, and not reached, respectively, for first-line IO-tyrosine kinase inhibitor therapy. In univariate and multivariate analyses, discontinuation of first-line treatment because of progressive disease and liver metastasis were independent risk factors for PFS. All-grade adverse events occurred in 72% of patients, and grade 3 or higher adverse events occurred in 28% of patients. Second line-cabozantinib after first-line IO combination therapy for advanced renal cell carcinoma was expected to be effective after either IO-IO or IO-TKI treatment and feasible in real-world practice.

## Introduction

The estimated number of new cases of kidney cancer globally was 431,288 in 2020, and the disease was linked to 179,368 deaths^1^. Currently, immuno-oncology (IO) combination therapy is commonly used as a systemic treatment for advanced renal cell carcinoma (RCC)^2–6^. The emergence of this strategy has resulted in changes in both first- and second-line therapy. A promising phase 3 trial assessing second-line therapy after IO combination therapy is underway, but the results have not yet been published^7^. The tyrosine kinase inhibitor (TKI) cabozantinib is frequently used in clinical trials and clinical practice in the first-line setting both as monotherapy and in combination with IO therapy. In fact, the phase 3 METEOR trial compared cabozantinib with everolimus in patients previously treated with TKI monotherapy^8^. However, there are little data assessing the use of this treatment after IO combination therapy despite its frequent clinical use. It is important to recognize the therapeutic outcomes of cabozantinib after IO combination therapy in real-world settings. Some new second-line treatments including combination regimens could be approved in the future. Such treatments might not be suitable for all patients, especially those at higher risk of adverse events (AEs). There is a need to confirm the efficacy and safety of cabozantinib after IO combination therapy, which has not yet been verified on a large scale, using large-scale real-world clinical data. It is also necessary to clarify the outcomes of sequential treatments after cabozantinib. In this study, we assessed the use of cabozantinib in the second-line setting after IO combination therapy using large-scale study data from the Japanese Urological Oncology Group (JUOG) study data. This study is novel in that it examines the therapeutic outcomes, evaluation of progression risk factor and outcomes of sequential treatments of cabozantinib after IO combination in a real clinical setting on a large scale.

## Methods

Thirty-five university hospitals and cancer centers participated in this large-scale retrospective study in Japan. Patients with advanced renal cancer who started second-line therapy after the approval of nivolumab plus ipilimumab in 2018 were eligible. We retrospectively analyzed data for patients who received second-line cabozantinib therapy after IO combination therapy. This study was approved by the institutional review board of Hamamatsu University School of Medicine (No. 22-008). The institutional review board of Hamamatsu University School of Medicine approved that this research was properly conducted in an opt-out format. All methods were performed in accordance with the relevant guidelines and regulations by including a statement.

IO combination therapy includes IO-IO and IO-TKI. In this study, IO-IO is nivolumab + ipilimumab and IO-TKI is avelumab or pembrolizumab + axitinib. Second-line cabozantinib progression was defined as progression after administration of cabozantinib. Death after administration of cabozantinib was defined as an OS event with reference to past similar reports^9^.

The following patient data were retrospectively collected from hospital records: age, sex, smoking status, Eastern Cooperative Oncology group performance status (ECOG PS), primary tumor diameter, first-line combination therapy regimen, nephrectomy, histology, presence of sarcomatoid change, IMDC risk in the first and second lines, Best overall response (BOR) in the first and second lines, second-line cabozantinib progression free survival (PFS) and overall survival (OS) from second-line, reason for discontinuing first-line IO combination therapy, duration between first-line combination therapy and second-line cabozantinib therapy, metastatic organ during second-line cabozantinib therapy, lactate dehydrogenase levels, albumin levels, estimated glomerular filtration rate (eGFR), C-reactive protein levels at the time of cabozantinib initiation and third-line PFS and overall survival OS from third-line. All AEs that occurred during cabozantinib administration were collected.

All statistical analyses were performed using JMP Pro 16.0.1 (SAS Institute, Cary, NC, USA). A two-sided P-value of < 0.05 denoted statistical significance. Intergroup comparisons were performed using the Mann–Whitney *U* test and χ^2^ test. Risk factors related to progression were analyzed by the Cox proportional hazards model and Kaplan–Meier method. Variables significant at P < 0.05 in univariate analysis were included in multivariate analysis.

## Results

In total, 254 patients were enrolled in the JUOG global study, of whom 118 were included in this analysis. In the first-line setting, 73 patients received IO-IO therapy, and 45 patients received IO-TKI therapy. IO-IO consisted of nivolumab plus ipilimumab in all cases. Ten patients in the IO-TKI group received avelumab plus axitinib, and 35 received pembrolizumab plus axitinib.

### Patients’ background

Table 1 presents the patients’ background data. The median patient age was 67 years. The median duration of observation from second-line cabozantinib initiation was 10.5 months. In total, 60% of patients underwent nephrectomy, and 76% had clear cell histology. The IMDC risk at the start of first-line therapy was favorable, intermediate, and poor in 13%, 54%, and 31% of patients, respectively. The objective response rate (ORR) in the first-line setting was 33%. Meanwhile, 77% of patients discontinued first-line treatment because of disease progression, whereas 23% discontinued treatment because of AEs. The period between first-line combination therapy and second-line cabozantinib therapy was 6.7 months in the entire population, 7.8 months in the IO-IO group, and 6.4 months in the IO-TKI group. There were no differences in background data between the IO-IO and IO-TKI groups.

**Table 1:** Patients’ characteristics.

	All patients n = 118	First-line IO-IO n = 73	First-line IO-TKI n = 45	P
Age at the time of second-line cabozantinib initiation, median (range)	67 (37–87)	67 (37–87)	67 (42–80)	0.7500
Sex				
Male	93 (79%)	55 (75%)	38 (84%)	0.2398
Female	25 (21%)	18 (25%)	7 (16%)	
Nephrectomy				
Positive	71 (60%)	40 (55%)	31 (69%)	0.1287
Negative	47 (40%)	33 (45%)	14 (31%)	
Histology				
Clear cell	90 (76%)	54 (74%)	36 (80%)	0.0555
Non-clear cell	22 (19%)	18 (25%)	4 (9%)	
Unknown	6 (5%)	1 (1%)	5 (11%)	
Sarcomatoid change				
Present	12 (10%)	7 (10%)	5 (11%)	0.7791
Absent	96 (81%)	60 (82%)	36 (80%)	
Unknown	10 (9%)	6 (8%)	4 (9%)	
IMDC risk at the time of first-line therapy				
Favorable	15 (13%)	6 (8%)	9 (20%)	0.4035^#^
Intermediate	64 (54%)	41 (56%)	23 (51%)	
Poor	37 (31%)	25 (34%)	12 (27%)	
Unknown	2 (2%)	1 (3%)	1 (2%)	
IMDC risk at the time of second-line therapy				
Favorable	12 (10%)	2 (3%)	10 (22%)	0.2145^#^
Intermediate	72 (61%)	47 (64%)	25 (56%)	
Poor	34 (29%)	24 (33%)	10 (22%)	
Unknown	0 (0%)	0 (0%)	0 (0%)	
BOR for first-line therapy				
CR	2 (2%)	2 (3%)	0 (0%)	0.1184^†^
PR	37 (31%)	18 (25%)	19 (42%)	
SD	48 (41%)	31 (42%)	17 (38%)	
PD	29 (25%)	20 (27%)	9 (20%)	
NE	2 (2%)	2 (3%)	0 (0%)	
Reason for first-line therapy discontinuation				
PD	91 (77%)	58 (79%)	33 (73%)	0.4421
AE	27 (23%)	15 (21%)	12 (27%)	
Duration between first-line and second-line therapy (months), median (range)	6.7 (0.7–48.3)	7.8 (0.7–37.6)	6.4 (1.4–48.3)	0.3336
Metastatic organ at the start of second-line therapy				
Lungs	74 (63%)	48 (66%)	26 (58%)	
Liver	26 (22%)	15 (21%)	11 (24%)	
Bone	36 (31%)	20 (27%)	16 (36%)	
Brain	6 (5%)	3 (4%)	3 (7%)	
Median observation period from the initiation of second-line therapy (months)	10.5	10.5	10.5	0.3257

*IMDC* International Metastatic RCC Database Consortium, *BOR* Best overall response, *CR* complete response, *PR* partial response, *SD* stable disease, *PD* progressive disease, *NE* not evaluable, *AE* adverse event, *IO* immuno-oncology, *TKI* tyrosine kinase inhibitor.

^#^Poor vs. favorable/intermediate.

^†^CR/PR vs. SD/PD.

### Efficacy of second-line cabozantinib

Table 2 presents the BOR, PFS, and OS by first-line therapy. Overall objective response rate and disease control rate (DCR) were 32% and 75%. The IO-TKI group had a slightly lower ORR than the IO-IO group (37% vs. 24%). The DCR was 77% in the IO-IO group, versus 71% in the IO-TKI group. The BOR was progressive disease (PD) in 18% of patients in the IO-IO group and 22% of patients in the IO-TKI group.

**Table 2:** Efficacy of second-line cabozantinib.

Parameter	Total n = 118	First-line IO-IO n = 73	First-line IO-TKI n = 45
Best overall response			
CR, n (%)	1 (1%)	0 (0%)	1 (2%)
PR, n (%)	37 (31%)	27 (37%)	10 (22%)
SD, n (%)	50 (42%)	29 (40%)	21 (47%)
PD, n (%)	23 (19%)	13 (18%)	10 (22%)
NE, n (%)	7 (6%)	4 (5%)	3 (7%)
ORR, %	32%	37%	24%
DCR, %	75%	77%	71%
PFS, median (months)	10.5	11.1	8.3
OS, median (months)	NR	NR	NR

*CR* complete response, *PR* partial response, *SD* stable disease, *PD* progressive disease, *NE* not evaluable, *ORR* overall response rate, *DCR* disease control rate, *IO* immuno-oncology, *TKI* tyrosine kinase inhibitor, *NR* not reached.

**Figure 1:**
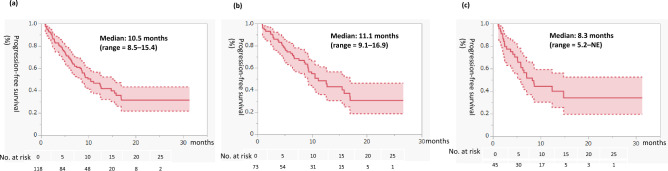
Progression-free survival for second-line cabozantinib after first-line immuno-oncology (IO) combination therapy. Rates were estimated using the Kaplan–Meier method. (**a**) All patients. (**b**) In patients who received first-line IO-IO therapy. (**c**) Patients who received first-line IO-tyrosine kinase inhibitor therapy. *NE* not evaluable.

**Figure 2:**
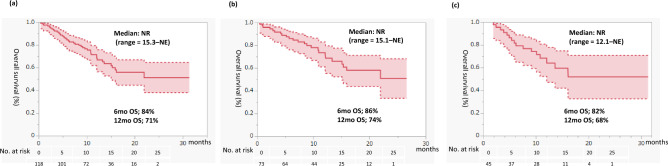
Overall survival for second-line cabozantinib after first line immuno-oncology (IO) combination therapy. Rates were estimated using the Kaplan–Meier method. (**a**) All patients. (**b**) In patients who received first-line IO-IO therapy. (**c**) Patients who received first-line IO-tyrosine kinase inhibitor therapy. *NR* not reached, *NE* not evaluable.

Figure 1 presents PFS for second-line cabozantinib treatment. PFS in the entire cohort was 10.5 months (95% confidence interval [CI] = 8.5–15.4). PFS in the IO-IO group was 11.1 months (95% CI = 9.1–16.9), compared with 8.3 months (95% CI = 5.2–not evaluable [NE]) in the IO-TKI group. Figure 2 presents OS for second-line cabozantinib treatment. OS for the total population was not reached (NR) (95% CI = 15.3–NE, 6-month OS rate was 84%, 12-month OS rate was 71%). OS was NR in both the IO-IO (95% CI = 15.1–NE, 6-month OS rate was 86%, 12-month OS rate was 74%) and IO-TKI groups (95% CI = 12.1–NE, 6-month OS rate was 82%, 12-month OS rate was 68%).

**Figure 3:**
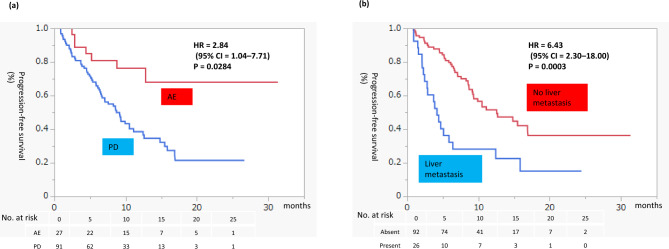
Progression-free survival for second-line cabozantinib after first-line immuno-oncology combination therapy based on independent risk factors. Rates were estimated using the Kaplan–Meier method. (**a**) Reason for first-line treatment discontinuation. (**b**) Presence of liver metastasis. *AE* adverse event, *PD* progressive disease, *HR* hazard ratio, *CI* confidence interval.

We examined the risk factors for PFS in all 118 patients (Table 3). According to univariate analysis, the reason for first-line treatment discontinuation (PD vs. AE: hazard ratio [HR] = 2.84, P = 0.0284), ECOG PS (0 vs. 1–2: HR = 0.35, P = 0.0157), and liver metastasis (present vs. absent: HR = 6.43, P = 0.0003) were risk factors for PFS. According to multivariate analysis, the reason for first-line treatment discontinuation and liver metastasis were independent risk factors for PFS. PFS according to these risk factors is presented in Fig. 3. Supplementary Fig. S1 presents PFS by reason for discontinuing each first-line regimen. In the IO-IO group, PFS tended to be worse for patients who discontinued treatment because of PD than for those who discontinued because of AEs.

**Table 3:** Results of univariate and multivariate analysis of risk factors for PFS.

Factor	Univariate model		Multivariate model	
	HR (95% CI)	P	HR (95% CI)	P
Age	0.38 (0.06–2.38)	0.3046		
Sex				
Male	0.44 (0.17–1.10)	0.0841		
Female				
Smoking				
Present	0.49 (0.22–1.07)	0.0742		
Absent				
Nephrectomy				
Present	1.32 (0.55–3.16)	0.5256		
Absent				
Histology				
Clear cell	0.99 (0.41–2.40)	0.9986		
Non-clear cell				
Sarcomatoid change				
Present	1.48 (0.49–4.49)	0.4885		
Absent				
Primary tumor diameter	1.69 (0.27–10.24)	0.5670		
First-line treatment				
IO-IO	0.47 (0.20–1.12)	0.0963		
IO-TKI				
Best response of first-line treatment				
CR or PR	0.74 (0.43–1.26)	0.2758		
SD or PD				
Reason for first-line treatment discontinuation				
PD	2.84 (1.04–7.71)	0.0284	4.40 (1.61–11.98)	0.0037
AE				
IMDC risk at the time of first-line treatment				
Favorable/intermediate	0.46 (0.17–1.26)	0.1361		
Poor				
Duration from first-line therapy to second-line cabozantinib	0.66 (0.04–6.33)	0.7443		
IMDC risk at the time of second-line treatment				
Favorable/intermediate	0.55 (0.18–1.66)	0.2935		
Poor				
ECOG PS				
0	0.35 (0.15–0.81)	0.0157	0.64 (0.27–1.51)	0.3134
1–2				
Metastatic organ				
Lungs				
Present	1.13 (0.50–2.54)	0.7594		
Absent				
Liver				
Present	6.43 (2.30–18.00)	0.0003	3.47 (1.20–9.99)	0.0207
Absent				
Bone				
Present	1.89 (0.80–4.47)	0.1500		
Absent				
Brain				
Present	0.46 (0.07–2.95)	0.3951		
Absent				
Lactate dehydrogenase	2.91 (0.20–21.92)	0.3731		
Albumin	1.98 (0.00–338.70)	0.8418		
Estimated glomerular filtration rate	0.47 (0.00–9.72)	0.6562		
C-reactive protein	0.70 (0.03–9.60)	0.7988		

*IO* immuno-oncology, *TKI* tyrosine kinase inhibitor, *PD* progressive disease, *AE* adverse event, *IMDC* International Metastatic RCC Database Consortium, *ECOG PS* Eastern Cooperative Oncology Group performance status, *HR* hazard ratio, *CI* confidence interval.

### Safety of second-line cabozantinib

AEs of any grade occurred in 72% of patients, whereas grade 3 or higher AEs occurred in 28% of patients (Table 4). Hypertension and hand–foot syndrome were the most frequent AEs, followed by liver dysfunction, fatigue, and diarrhea. There were no Grade 5 AEs. Select AEs are presented in Supplementary Table S1.

**Table 4:** Adverse events of second-line cabozantinib.

Events	Any grade (%)	Grade ≥ 3 (%)
Any adverse events	85 (72%)	33 (28%)
Hypertension	25 (21%)	8 (7%)
Hand–foot syndrome	25 (21%)	3 (3%)
Liver dysfunction	16 (14%)	6 (5%)
Fatigue	16 (14%)	3 (3%)
Diarrhea	15 (13%)	1 (1%)
Stomatitis	12 (10%)	2 (2%)
Hypothyroidism	10 (8%)	0
Proteinuria	6 (5%)	3 (3%)
Rash	6 (5%)	2 (2%)
Appetite loss	5 (4%)	1 (1%)
Taste disorder	5 (4%)	0
Gastrointestinal bleeding	3 (3%)	2 (2%)
Thrombocytopenia	3 (3%)	0
Nausea	2 (2%)	0
Headache	2 (2%)	0
Hoarseness	2 (2%)	0

### Efficacy of third-line treatment after cabozantinib

**Figure 4:**
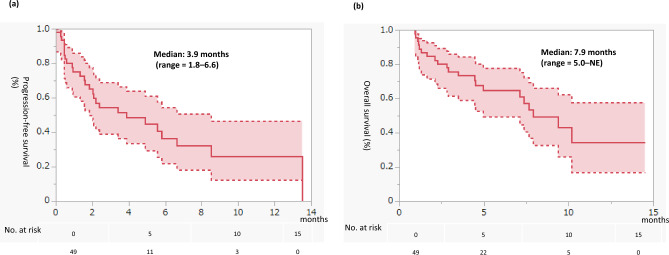
Progression-free survival (PFS) and overall survival (OS) for third-line treatment after second-line cabozantinib. Rates were estimated using the Kaplan–Meier method. (**a**) PFS. (**b**) OS.

Finally, we analyzed PFS and OS in the third-line setting after second-line cabozantinib treatment in 49 patients (Fig. 4). The third-line therapy was axitinib in 19 patients, nivolumab in 12 patients, pazopanib in 6 patients, sunitinib in 5 patients, everolimus in 3 patients, temsirolimus in 1 patient, and others in 3 patients. In this group, PFS was 3.9 months (95% CI = 1.8–6.6), whereas OS was 7.9 months (95% CI = 5.0–NE).

### Institutional review board statement

This study was approved by the Institutional Review Board of Hamamatsu Medical University Hospital (IRB No. 22-008).

### Informed consent

Informed consent was obtained by the opt-out method in the study. The requirement for patient consent was waived because this was a retrospective review of medical practice covered by health insurance.

## Discussion

In this study, we analyzed the actual clinical situation of cabozantinib as a second-line treatment after IO combination therapy using large-scale retrospective data from JUOG. Although this study included a large cohort and the treatment strategies slightly differed at each facility, it is meaningful to investigate the actual situation of second-line treatment after IO combination treatment in Japan in recent years. The purpose of this study was to clarify the utility of second-line cabozantinib treatment after IO combination therapy. The ORR for second-line cabozantinib after IO combination therapy was 32%, PFS was 10.5 months, and OS was NR (6-month OS rate was 84%, 12-month OS rate was 71%). The factors significantly associated with efficacy were first-line treatment discontinuation because of PD and liver metastasis. None of the AEs significantly exceeded those previously reported. Sequential treatment remains important for metastatic RCC treatment. Third-line treatment after cabozantinib administration was associated with PFS and OS of 3.9 and 7.9 months, respectively, indicating that efficacy can be expected after cabozantinib treatment.

Based on the results of the phase 3 METEOR trial^8^, we are using cabozantinib in clinical practice. Compared with everolimus, cabozantinib provided clear benefits for the primary endpoint of PFS and secondary endpoint of OS. The AEs of this drug were also manageable. However, in METEOR trial, approximately 5% of patient received IO before cabozantinib. It is unlikely that this situation reflects the efficacy of cabozantinib after IO administration.

The recently reported phase 3 CONTACT-03 trial evaluated the efficacy of cabozantinib in patients previously treated with IO^10^. This trial compared the efficacy of cabozantinib alone and in combination with atezolizumab. First, it should be noted that the cabozantinib monotherapy group had median PFS of 10.8 months, in line with the current study results. The results support the efficacy of cabozantinib as sequential treatment in the IO era. Median OS was NR. The results of this phase 3 trial failed to demonstrate the efficacy of add-on atezolizumab. The BREAKPOINT trial, a phase 2 study in 31 cases has been reported. Median PFS was 8.3 months, OS was 13.8 months and ORR was 37.9%. Grade 3–4 adverse events occurred in 47%. Although this study involved a relatively small number of cases, it is a very important result when considering the theme of this paper^9^.

The After-IO trial was conducted as a follow-up study of a phase 3 trial of patients treated with nivolumab^11^. Axitinib and other TKIs have produced high response rates. However, information on cabozantinib was lacking in this study because of the timing of its approval. A report from the Italian Meet Uro 7 group provided real-world results for cabozantinib after IO monotherapy^12^. According to this study, cabozantinib was linked to longer PFS than everolimus and other TKIs. In addition, cabozantinib is the most frequently selected drug in clinical practice. Thus, the results of the Italian study do not reflect the efficacy of cabozantinib in clinical practice after IO combination therapy, which is currently the mainstream strategy.

The reason for first-line treatment discontinuation was an independent predictor of PFS in patients treated with cabozantinib. In patients who discontinued first-line therapy because of AEs, median PFS was not reached in the IO-IO or IO-TKI group. We believe that cabozantinib administration in a state of relatively high drug sensitivity led to good results. The time from first-line treatment to cabozantinib administration was not a significant predictor of PFS. In this regard, some patients are expected to be highly sensitive to drug therapy in general. Conversely, second-line treatment is not effective in some patients with rapid tumor growth and a short period between first- and second-line treatment. We anticipate that the efficacy of cabozantinib will be limited in some patients with accelerated tumor growth after PD. Median PFS did not reach 10 months in patients who discontinued first-line treatment because of PD, highlighting the need for new systemic drugs.

Analyses have been conducted by metastatic organ in patients with RCC. Metastasis to the liver, bone, and brain is associated with poor prognoses. Xue et al. also found that liver metastases carried the worst prognosis^13^. In our study, the efficacy of cabozantinib in patients with bone and brain metastases was relatively satisfactory. Cabozantinib has inhibitory effects on MET and AXL, and it is expected to be effective against bone metastasis^14^. The METEOR trial also recorded a high response rate in patients with bone metastasis^8^. Cabozantinib exhibited considerable intracranial activity and an acceptable safety profile in patients with RCC and brain metastases^15^. In this study, we were unable to examine the details of local treatment of the brain. In addition, few cases of brain metastasis have been investigated, and further analysis is required. A certain effect has been demonstrated, as indicated by the results of this study. It is suggested that efficacy can be expected even after IO combination treatment in these metastatic organs. Meanwhile, the efficacy in patients with liver metastasis was limited. Liver metastases from RCC are reported to carry a poorer prognosis than liver metastases from other cancer types treated with IO^16^. Several reports discussed poor prognosis associated with liver metastases. James et al. found that the presence of liver metastasis significantly reduced tumor-specific immunity in an antigen-specific, PD-1-dependent manner. This process was associated with the coordinated activation of regulatory T cells and modulation of intratumoral CD11b+ monocytes^17^. The presence of liver metastasis was correlated with fewer CD8+ T cells at the invasive margin in distant tumors^18^. We expect new systemic treatments in the future for patients with liver metastasis.

The evaluation of AEs is expected to differ between retrospective studies and clinical trials. In addition, this study was based on multi-institutional data, and there are disparities in the awareness of AEs among institutions. The rate of all-grade AEs was somewhat low compared with the findings for cabozantinib in clinical trials^8^. This finding should not be interpreted as a low incidence of AEs in the Japanese population but rather as a limitation of information collection. However, the incidence of grade 3 or higher AEs was 28%, in line with prior findings^8^. These data serve as an index for Japanese data after IO combination treatment. No grade 5 AEs were observed in this population. It is considered that cabozantinib can be used safely in this population after molecular targeted therapy. A report found that Japanese patients are relatively prone to liver dysfunction^19^, and the present data recorded Grade 3 or higher liver dysfunction in six patients, which should be noted.

Expectations for sequential therapy might be lower than that in the previous age of molecular targeted drugs, but in real-world practice, sequential systemic therapy is often used to treat metastatic RCC. When performing sequential treatment, it is desirable to avoid the situation in which subsequent therapeutic effects are not anticipated. Cabozantinib is a relatively effective TKI, and the effects of subsequent systemic treatment after cabozantinib were analyzed in this study. Although PFS and OS were not substantially extended, a certain effect can be expected in patients who started third-line treatment. Luigi et al. summarized systemic treatment after cabozantinib in 56 patients. Median OS after cabozantinib was 7.7 months, while median TTF after cabozantinib was 2.8 months. However, only three of the participants in this study used IO combination as first-line treatment^20^. There are no large-scale reports of the use of cabozantinib after IO combination followed by systemic treatment. Of course, some patients receive best supportive care after cabozantinib administration, suggesting that AE management is possible.

Several combination treatments have been reported as second-line treatments after IO combination in recent years. One study verified the effect of adding atezolizumab to cabozantinib^10^. Unfortunately, no benefit was observed. Another phase 2 single-arm trial examined combination therapy with cabozantinib and belzutifan, in which PFS was 13.8 months^21^. Although a simple comparison cannot be made, the addition of belzutifan could be promising given the PFS of approximately 10 months in our study and the aforementioned CONTACT03 trial^10^. The phase 3 LITESPARK011 trial comparing cabozantinib with the combination of lenvatinib and belzutifan is currently underway^7^. The result of this study should be watched closely. It is hoped that the approval of these promising treatments will lead to improved prognoses in patients who discontinued IO combination therapy because of PD and patients with liver metastases, who had poor prognoses in this study.

This research had some limitations. First, this was a retrospective study. Treatment selection was left to the discretion of each facility, leading to varied treatments. In addition, this research used information from facilities such as university hospitals and cancer centers, and there is a possibility that the protocols of these situations slightly deviate from those used in hospitals throughout Japan. At the time of enrollment in this study, no patients in whom lenvatinib plus pembrolizumab was discontinued and cabozantinib was administered were included. In actual clinical practice, this strategy is frequently employed. New research is expected to clarify this issue in the future. We were not able to verify the effects of cabozantinib at different starting doses in this study. This is an issue that should be verified in future research. Phase II CaboPoint trial is now on-going^22^. It is hoped that the results will become clearer once the results of this trial are published.

In this study, we analyzed the real-world data of cabozantinib in Japan after IO combination therapy using the JUOG database. A relatively high response rate was obtained even after IO combination therapy, and AE management was possible. The results of this relatively large-scale study clarified the usefulness of cabozantinib and identified factors associated with poor efficacy, namely first-line treatment discontinuation because of PD and liver metastasis.

## Conclusions

To the best of our knowledge, this is the first study to evaluate the efficacy and safety of second-line cabozantinib therapy after first-line IO combination treatment for advanced RCC using a Japanese multi-institutional database. ORR was 32%, DCR was 75%, median PFS was 10.5 months, and median OS was NR. No adverse events were reported after IO combination therapy.

### Supplementary Information

Supplementary Information.

## Data Availability

The data presented in this study are available on request from the corresponding author.
