# Supplementary material for: Efficacy and safety of second-line cabozantinib after immuno-oncology combination therapy for advanced renal cell carcinoma: Japanese multicenter retrospective study
Source: Sci Rep. 2023 Nov 23;13:20629. doi: 10.1038/s41598-023-48087-4 (PMC10667220; doi:10.1038/s41598-023-48087-4)
Supplement: Supplementary file 1 — Supplementary Information. [file 41598_2023_48087_MOESM1_ESM.pptx]

## Slide 1
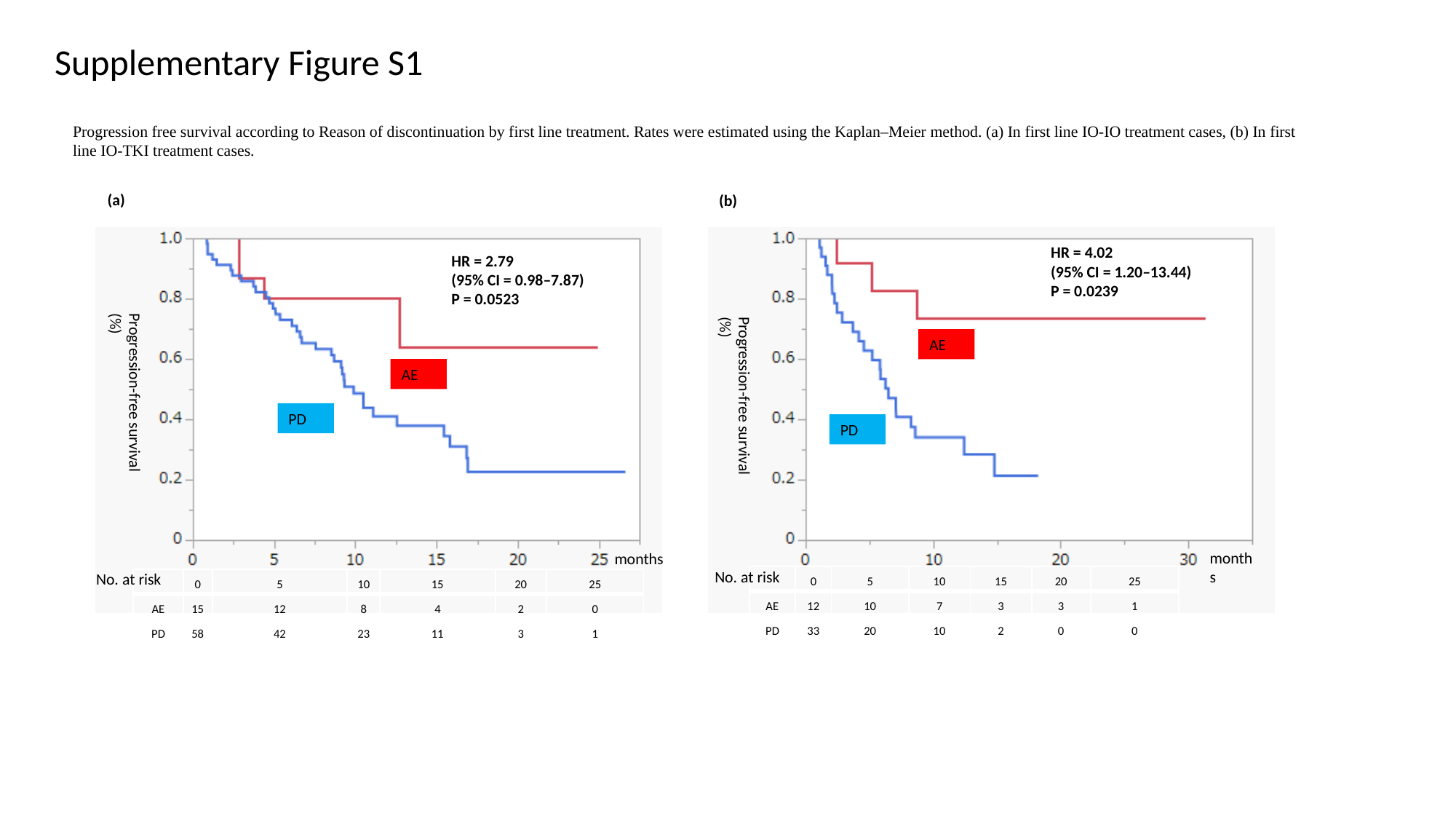

Supplementary Figure S1
Progression free survival according to Reason of discontinuation by first line treatment. Rates were estimated using the Kaplan–Meier method. (a) In first line IO-IO treatment cases, (b) In first line IO-TKI treatment cases.
(a)
(b)
HR = 4.02
(95% CI = 1.20–13.44)
P = 0.0239
HR = 2.79
(95% CI = 0.98–7.87)
P = 0.0523
Progression-free survival (%)
Progression-free survival (%)
AE
AE
PD
PD
months
months
No. at risk
No. at risk
| | 0 | 5 | 10 | 15 | 20 | 25 |
| --- | --- | --- | --- | --- | --- | --- |
| AE | 12 | 10 | 7 | 3 | 3 | 1 |
| PD | 33 | 20 | 10 | 2 | 0 | 0 |
| | 0 | 5 | 10 | 15 | 20 | 25 |
| --- | --- | --- | --- | --- | --- | --- |
| AE | 15 | 12 | 8 | 4 | 2 | 0 |
| PD | 58 | 42 | 23 | 11 | 3 | 1 |

## Slide 2
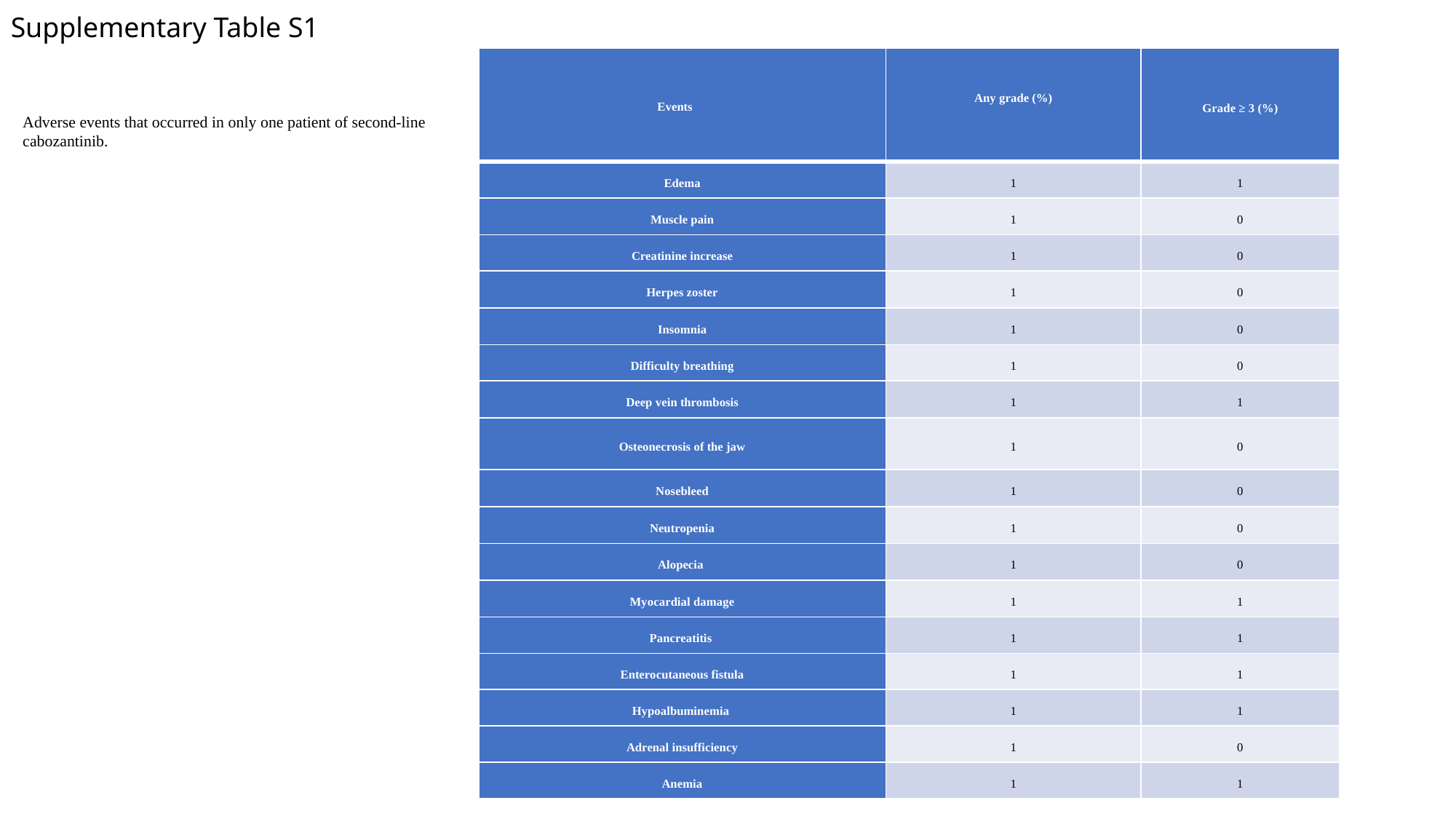

Supplementary Table S1
| Events | Any grade (%) | Grade ≥ 3 (%) |
| --- | --- | --- |
| Edema | 1 | 1 |
| Muscle pain | 1 | 0 |
| Creatinine increase | 1 | 0 |
| Herpes zoster | 1 | 0 |
| Insomnia | 1 | 0 |
| Difficulty breathing | 1 | 0 |
| Deep vein thrombosis | 1 | 1 |
| Osteonecrosis of the jaw | 1 | 0 |
| Nosebleed | 1 | 0 |
| Neutropenia | 1 | 0 |
| Alopecia | 1 | 0 |
| Myocardial damage | 1 | 1 |
| Pancreatitis | 1 | 1 |
| Enterocutaneous fistula | 1 | 1 |
| Hypoalbuminemia | 1 | 1 |
| Adrenal insufficiency | 1 | 0 |
| Anemia | 1 | 1 |
Adverse events that occurred in only one patient of second-line cabozantinib.
